# Swedish translation and cross-cultural adaptation of eight pediatric item banks from the Patient-Reported Outcomes Measurement Information System (PROMIS)^®^

**DOI:** 10.1186/s41687-021-00353-7

**Published:** 2021-09-06

**Authors:** Ida Blomqvist, John Eric Chaplin, Evalill Nilsson, Eva Henje, Inga Dennhag

**Affiliations:** 1 Department of Clinical Science, Child- and Adolescent Psychiatry, Umeå University, 90185 Umeå, Sweden; 2 Department of Pediatrics, Institute of Clinical Sciences, University of Gothenburg, Gothenburg, Sweden; 3 Department of Medicine and Optometry, Linnaeus University, Kalmar, Sweden

**Keywords:** Pediatric PROMIS, Translation, Linguistic validation, Cultural adaptation, Questionnaire, Item bank

## Abstract

**Background:**

This study is part of the Swedish initiative for the establishment of standardized, modern patient-reported measures for national use in Swedish healthcare. The goal was to translate and culturally adapt eight pediatric Patient-Reported Outcomes Measurement Information System (PROMIS^®^) item banks (anger, anxiety, depressive symptoms, family relationships, fatigue, pain interference, peer relationships and physical activity) into Swedish.

**Methods:**

Authorization to translate all currently available pediatric PROMIS item banks (autumn, 2016) into Swedish was obtained from the PROMIS Health Organization. The translation followed the Functional Assessment of Chronic Illness Therapy translation recommendations with one major modification, which was the use of a bilingual multi-professional review workshop. The following steps were applied: translation, reconciliation, a two-day multi professional reviewer workshop, back translation, and cognitive debriefing with eleven children (8–17 years) before final review. The bilingual multi-professional review workshop provided a simultaneous, in-depth assessment from different professionals. The group consisted of questionnaire design experts, researchers experienced in using patient-reported measures in healthcare, linguists, and pediatric healthcare professionals.

**Results:**

All item banks had translation issues that needed to be resolved. Twenty-four items (20.7%) needed resolution at the final review stage after cognitive debriefing. The issues with translations included 1. Lack of matching definitions with items across languages (6 items); 2. Problems related to language, vocabulary, and cultural differences (6 items); and 3. Difficulties in adaptation to age-appropriate language (12 items).

**Conclusions:**

The translated and adapted versions of the eight Swedish pediatric PROMIS item banks are linguistically acceptable. The next stage will be cross-cultural validation studies in Sweden. Despite the fact that there are cultural differences between Sweden and the United States, our translation processes have successfully managed to address all issues. Expert review groups from already-established networks and processes regarding pediatric healthcare throughout the country will facilitate the future implementation of pediatric PROMIS item banks in Sweden.

## Background

The Patient Reported Outcome Measurement Information System (PROMIS) was established in 2004 with funding from the US National Institutes of Health (NIH), with the goal to “develop an efficient state-of-the-art assessment system for self-reported health.” PROMIS has now been used in research and clinical practice for over a decade, mostly with adult patients e.g. [5], but also with children and their parents (e.g. [7–9, 14, 16, 21].

PROMIS has been shown to have higher reliability, validity, and better sensitivity to symptom change than most legacy health measures [2, 4, 5, 12]. PROMIS uses item banks instead of traditional questionnaires. An item bank is a set of questions which measure the same underlying construct. One advantage of the item bank format is that the items can be presented to the respondent through computerized adaptive testing (CAT) [3, 19]. CAT adapts uniquely to each respondent based on previous responses. This method iteratively selects the most suitable items to complete to reach a high precision, which provides both precise and adequate information, and often results in fewer items to be answered and a reduced burden for the respondent.

The PROMIS system has a rapidly growing global coverage [2, 5], including Sweden [6, 17, 20]. PROMIS is utilized for a variety of clinical conditions, e.g., cancer, rheumatism, asthma, and sickle-cell disease [7, 13, 16, 21, 25].

In Sweden, a project to translate and culturally adapt PROMIS item banks to Swedish, including the pediatric banks, was launched in 2016. In Sweden, pediatric health questionnaires are widely used, but several have not been properly validated [22]. This study is part of the Swedish initiative to establish standardized modern patient reported measures for national use in Swedish healthcare, and aims to create a shared unified terminology and metric for reporting on common symptoms and functional life domains.

The main purpose of this study was to improve equity and quality of assessment and monitoring of health service delivery for children and adolescents with various medical conditions and psychiatric disorders by translating and culturally adapting eight pediatric PROMIS item banks for Swedish use.

## Method

Authorization to translate the eight PROMIS pediatric item banks to Swedish was obtained from PROMIS Health Organization (PHO) in 2016 (see Table 1 for an overview). All items have unipolar verbal response scales, for example, the Depressive symptoms item banks provide five response alternatives: never, almost never, sometimes, often, almost always. The PROMIS organization offers pediatric item definition lists to aid translations by clarifying the concept behind each item [18]. Item definition lists were available for the following six out of eight item banks: Anger, Anxiety, Depressive symptom, Fatigue, Pain interference, and Peer relationships (definition lists were lacking for Family relationships and Physical activity).

**Table 1:** An overview of the pediatric PROMIS item banks translated to Swedish

	English	Swedish	Items
Physical health	PROMIS Pediatric Bank v2.0—Pain interference	Smärtpåverkan	20
	PROMIS Pediatric Bank v2.0—Fatigue	Trötthet	25
	PROMIS Pediatric Bank v1.0—Physical activity	Fysisk aktivitet	10
Mental health	PROMIS Pediatric Bank v2.0—Depressive symptom	Depressiva symptom	14
	PROMIS Pediatric Bank v2.0—Anxiety	Ångest	15
	PROMIS Pediatric Scale v2.0—Anger 9a	Ilska	9
Social health	PROMIS Pediatric Bank v2.0 – Peer relationships	Kamratrelationer	15
	PROMIS Pediatric Short Form v.1.0—Family relationships 8a	Familjerelationer	8

The translation process followed the Functional Assessment of Chronic Illness Therapy (FACIT) translation methodology [11] with some modifications (see Table 2), and semantic/linguistic, content, and conceptual adaptation was performed [24]. After two independent forward translations were completed, any differences in the two translations were reconciled by the original translators and a third researcher. This version of the translation was then submitted to a multi-professional bi-lingual review group. This review group (a modification of the FACIT method [11], provided an in-depth assessment by different professionals simultaneously. The group consisted of questionnaire design experts, researchers experienced in using patient-reported measures in healthcare, linguists, and pediatric healthcare professionals. In total, 21 people from different geographical regions of Sweden participated. The group review was conducted in a two-day session which started with general information about PROMIS, test adaptation, and translation methodology. The group was then divided into four smaller subgroups, each responsible for reviewing 4–6 item banks. Individual reviewing by each group member was conducted before group discussions. Back translation was then carried out and a final review was made by the bilingual translation team. A member of the PROMIS organization (JC) reviewed each back-translated item to assess the equivalence of the source and target translation. A final report was written to document the development of each translation.

**Table 2:** Translation process (modified FACIT methodology)

Steps in the process	Description
1. Forward translation	Two translators with experience of health questionnaires independently translated into Swedish
2. Reconciliation	Reconciliator with expert knowledge in health questionnaire design, aiming to resolve discrepancies in the translations and adapt to modern questionnaire design requirements
3. Expert reviews	First independently and then in small groups of 3 to 5 people examined the linguistic adequacy and meaning equivalence of the reconciled item translation. With reference to professional knowledge of the theoretical construct and the definition
4. Back-translation	Back translation and comparison with the original English item banks was done by a bilingual native English speaker
5. Cognitive debriefing	Eleven healthy adolescents between 8 and 17 years (9 girls and 2 boys, mean age 14 years, median age 14 years) completed the cognitive debriefing individually. The participants were interviewed by four interviewers
6. Final report	A report was written at the end of the process, documenting the development of the translation

### Cognitive debriefing interviews

Cognitive debriefing interviews were carried out for all eight item banks by four researchers specifically trained in conducting these interviews. A cognitive interview manual was written in Swedish for guidance. The interviews were performed with eleven children of varied ages and gender (see Table 2). All children were fluent in Swedish. Think-aloud methodology and subsequent respondent debriefing were used [15]. In this approach, respondents are requested to think out loud while answering five to six questions (depending on the item bank), followed by a short interview about the relevant items.

The pre-final version of the questionnaire was completed by a small sample of the target population. All respondents received the item banks in the same order. Comprehensibility, relevance, acceptability, and feasibility of all questions were considered [24]. The respondents answered the full battery of questions over the Internet and were then asked how they perceived the questions and the on-line survey.

### Data analyses

Both inductive and deductive methodologies (content analysis as per [23] were used to analyze the data qualitatively and simultaneously quantify the data. The number of items that belonged to each subtheme were counted, and it was noted when in the translation process the potential issues were found and how many times.

## Results

Eight pediatric PROMIS item banks were translated to Swedish, and all of them had translation issues to be resolved. In total, the eight item banks consisted of 116 items, and 24 of these items (20.7%) were problematic for translation and required specific linguistic attention.

The translation problems were categorized into three themes: 1. Lack of matching definitions with items across languages (6 items), 2. Problems related to language, vocabulary, and cultural differences (6 items), and 3. Difficulties in adaption to age-appropriate language (12 items). Table 3 presents the themes and subthemes of translations issues with examples.

**Table 3:** Themes of translation issues

Main themes	Subthemes	Example of issues (number of items)
Lack of matching definitions with items across languages	Equivocal items with precise definitions	Anxiety item bank: ‘I was afraid of going to school’, where the item could mean both being in school and traveling to school. The item was translated in accordance to the item definition^a^ and the concept of being in school. (5)
	Equivocal items without precise definitions	Physical activity item bank: ‘How many days did you run for 10 min or more?’. Whether it refers to 10 min of continuous running or 10 min in total during the day is not clear. The item was translated to 10 min of continuous running. (1)
Problems related to language, vocabulary and cultural differences	Adjectival agreement on intensity levels of the concept to be translated	Anxiety item bank: The translation of the word ‘scared’ used in the Anxiety item bank was translated to ‘rädd’. The word ‘rädd’, can be back-translated to either ‘scared’ or ‘afraid’. In Swedish, the grade difference between afraid and scared is more difficult to clearly illustrate using only a single word. (2)
	Culturally specific idiomatic phrases	Physical activity item bank: ‘How many days did you exercise or play so hard that your muscles burned?’. ‘Muscles burned’ is an idiomatic phrase in English and this could not be translated directly to Swedish. Instead, this was translated to ‘How many days did you exercise or play so much that you got aching muscles?’. (3)
	Cultural differences of measurements	Pain interference item bank: ‘It was hard for me to walk one block when I had pain’. The informal American English measurement of ‘one block’ has no Swedish equivalent. The translation must therefore relate to either the exact distance of a block (if such an exact measure exists) or an approximation. The Swedish translation used both: ‘it was difficult for me to walk a short distance (about 100 m) when I was in pain’. (1)
Difficulties in adaption to age-appropriate language	Comprehensibility of the items changes in the translation process	Anger item bank: I felt upset’ the most precise translation of the item ‘I felt upset’ in the Anger item bank was ‘upprörd’ which was difficult to understand for children of younger age. Hence, this item was instead translated to ‘I felt both angry and sad’. (4)
	Acceptance of the items for all age groups	Peer relationships item bank: ‘Other kids wanted to be with me’ was not accepted by teenagers until ‘kids’ was replaced by ‘others my age’. This phrase was then reused in other items for consistency and thus avoided the problem of having to use the word ‘child’. (8)

^a^Definition list from PROMIS organization [18]

### Lack of matching definitions with items across languages

We identified two subthemes of translation issues related to matching definitions with items across languages: 1. *Equivocal items with precise definitions*, i.e., the Swedish translation was more in accordance with the item definition in the definition list than the original English version; 2. *Equivocal items without precise definitions*, i.e., a lack of adequate definitions in the definition list made the item difficult to translate with precision.

### Problems related to language, vocabulary, and cultural differences

We identified three subthemes of translation issues: 1. *Adjectival agreement on intensity levels of the concept to be translated*; here, the differing availability of appropriate Swedish adjectives had to be overcome; 2. *Culturally-specific idiomatic phrases* made nine items difficult to translate; 3. *Cultural differences of measurements*; i.e., distance, weights, and volume are measured differently both formally and informally in the two nations, which presented a problem in the translation process.

### Difficulties in adaption to age-appropriate language

We identified two subthemes of translation issues: 1. *Comprehensibility of the items changes in the translation process*; an effort was made to harmonize items between age groups and make it comprehensive for all ages of respondents; 2. *Acceptance of the items for all age groups*; for example, the word “kid” was avoided in order to make the translation more acceptable for teenagers.

## Discussion

Eight pediatric PROMIS item banks containing 116 items were translated to Swedish, out of which 24 items presented translational problems that needed to be resolved. After thorough adaptation and translation, using internationally standardized methods, acceptable translations were obtained for all items and were sent for further processing to the PROMIS organization. In our translation process, we identified some potential issues, including problems of cultural non-equivalencies.

First, it should be noted that the PROMIS organization provides item bank definitions for some but not all item banks. These definition lists were vitally important for the translation process and may even have led to greater precision in the meaning of an item as compared to the original English version.

Second, a general effort has been made in many pediatric questionnaires to include items perceived as relevant across the whole relevant age range, which is usually done in order to simplify administration of the questionnaires which otherwise would need multiple age range versions to reflect maturation of language, vocabulary, and developmental stages. Generic terms such as ‘child’ or ‘kid’ might be adequate to use even for teenagers in some languages, but in other languages, such as Swedish, it is not appropriate. The items in the pediatric banks are intended to be suitable for both children and adolescents (8–17 years), therefore, we replaced ‘kids’ with ‘others my age’.

Third, the hierarchy of the adjectives (e.g., ‘afraid’ and ‘scared’) is hard to retain without reference to a standardized calibration of the item location [1]. In the lack of such a reference, we used the item definition list [18].

Lastly, the translation process also led to decisions similar to those made in other language translations [8, 14] regarding measures of distance, time, and idiomatic phrases. Measures often need context and qualitative descriptions to be understandable. Idiomatic phrases are often difficult to understand and should be avoided.

## Limitations

A possible limitation was that the cognitive debriefing took place in a single geographical area; hence, there is a risk of not reflecting the dialectal variations of the country. Richer data might have been obtained by including a larger representation of boys, younger children, and children from more diverse linguistic groups in the cognitive debriefing.

Our next step is to validate the item banks in community and patient samples, develop CAT, and perform differential item functioning (DIF) between English and Swedish in addition to other languages. The investigation of language-related DIF will enable us to evaluate the translation and cultural adaption process. With this knowledge, an additional goal will be to translate and culturally adapt more of the pediatric PROMIS item banks for children and parents into Swedish.

Using patient representatives in the development of the items is part of the philosophy of PROMIS (DeWalt, Rothrock, Yount, Stone, & Group, 2007). In the previous translation of the adult PROMIS items into Swedish, patient representatives were included in the review groups, which was beneficial to the whole process. The intention for future studies is to include children, adolescents, and caregivers in a similar way.

## Conclusions

In conclusion, the translated and adapted versions of the eight Swedish pediatric PROMIS item banks are linguistically acceptable. Despite the fact that there are cultural differences between Sweden and the United States, our translation processes have successfully addressed the relevant issues.

In our study, we utilized the FACIT methodology for translation with some modifications. Expert review groups emanating from already established networks and processes regarding pediatric healthcare throughout the country will facilitate the future implementation of pediatric PROMIS item banks in Sweden.

## Data Availability

The datasets used and/or analyzed during the current study are available from the corresponding author on reasonable request.

## Abbreviations

CAT: Computerized adaptive testing
DIF: Differential item functioning
FACIT: Functional assessment of chronic illness therapy
IRT: Item response theory
NIH: National Institute of Health
PHO: PROMIS Health Organization
PROMIS: Patient-Reported Outcomes Measurement Information System
PROs: Patient-reported outcomes
